# Whispers of hunger: a journey through anorexia nervosa

**DOI:** 10.1192/j.eurpsy.2025.1163

**Published:** 2025-08-26

**Authors:** A. Monllor, M. Rios, G. Lorenzo Chapatte, L. Rojas Vazquez, G. Guerra Valera, P. Marques Cabezas, M. Queipo de Llano de la Viuda, M. Andreo Vidal, M. Calvo Valcarcel, M. P. Pando Fernandez, P. Martinez Gimeno, B. Rodriguez Rodriguez, N. Navarro Barriga, M. Fernandez Lozano, M. J. Mateos Sexmero

**Affiliations:** 1 Hospital Clínico de Valladolid; 2 Hospital Clínico Valladolid, Valladolid, Spain

## Abstract

**Introduction:**

A 13 years old girl, who has always been an A* student, was brought to our Emergency Room, referred by her Psiquiatrist, to be hospitalized in our Unit.

During the first interview in the ER, the patient reported that she refused to eat food that had empty calories, which were essentially everything except water, and even water at times was an issue. She had a IMC of 16,57 kg/m2.

**Objectives:**

The objetive of this case is to try and explain how serious of a disorder anorexia nervosa can be, at times severe enough to distort completely the patients reality.

**Methods:**

The following patient will be presented, doing a thorough systematic bibliography review.

**Results:**

The patient remains admitted to our unit. After more than one month and a half, since first being admitted, the patient has both verbalized wanting to end her life before gaining weight, as well as improved her disorder awareness.

At first, she was bluntly negative to eat any food, to a point to such extent, that feeding via nasogastric tube was necessary.

After daily interviews, along with many consultations, both with clinical psychology and psychiatry, using both cognitive behavioral psychotherapy and psychotrpic drugs; we achieved a slight improvement by eating half plates of both first and second plate, as well as dessert of a 1300-1500kcal diet.

Nonetheless, the patient still manifest up to this day, her willingness of not to eat and to end her life if its necessary to avoid eating.

**Conclusions:**

Given the severity of the eating disorder suffered by the patient, social work was notified of the refferral to a long-term center specialized in eating disorders. The patient verbalized not wanting to got there, but recognized her disorder was getting more severe and the need for a long-term Unit.

**Disclosure of Interest:**

None Declared

